# The ICF Classification System to Assess Risk Factors for CVD in Secondary Prevention after Ischemic Stroke and Intracerebral Hemorrhage

**DOI:** 10.3390/medicina57030190

**Published:** 2021-02-24

**Authors:** Mateusz Lucki, Ewa Chlebuś, Agnieszka Wareńczak, Przemysław Lisiński

**Affiliations:** 1 Department of Cardiology, Hospital Center of the Jelenia Góra Valley, 58-506 Jelenia Góra, Poland; 2 Department of Rehabilitation and Physiotherapy, University of Medical Sciences, 60-545 Poznań, Poland; ewachlebus@ump.edu.pl (E.C.); agnieszka.warenczak@gmail.com (A.W.); plisinski@vp.pl (P.L.)

**Keywords:** stroke, CVD risk factors, secondary prevention, ICF

## Abstract

*Background and objectives*: Patients with a history of prior stroke have a high risk for subsequent cardiovascular events (CVD). Therefore, the implementation of an effective strategy to reduce risk factors and thereby improve secondary prevention outcomes is crucial in this patient population. The aim of this study was to determine differences in the incidence of risk factors for recurrent CVD events based on clinical type of prior stroke and to characterize them using the ICF (International Classification of Functioning, Disability and Health) classification system. *Materials and Methods*: The incidence of risk factors for recurrent CVD events were retrospectively analyzed in 109 patients with a history of ischemic stroke (IS) and 80 patients with a history of intracerebral hemorrhage (ICH) within 14 days poststroke. *Results*: Atrial fibrillation/flutter (*p* = 0.031), >70% carotid artery stenosis (*p* = 0.004), blood pressure >140/90 mmHg (*p* = 0.025), blood HbA1c levels >7% (*p* = 0.002), smoking (*p* = 0.026) and NSAID (nonsteroidal anti-inflammatory drug) use (*p* < 0.001) were significantly more common in patients with a history of ischemic stroke. However, liver function test abnormalities were observed more commonly in patients with a history of hemorrhagic stroke (*p* = 0.025). *Conclusions*: The incidence and type of risk factors for recurrent CVD events vary according to the clinical type of prior stroke. The ICF classification system is a useful tool for evaluating these risk factors. This may help reduce the risk of subsequent CVD events.

## 1. Introduction

Patients with a history of prior stroke have a high risk for subsequent cardiovascular events (CVD) [1]. The risk of recurrence is 1.7–4% in the 30 days after the first episode, 6–12% in the first year, and 19–42% in the next 5 years after the first stroke. The prognostic factors for a recurrent stroke depend on the time after the first stroke. There are risk factors for early recurrence (up to 30 days) and late recurrence (30 days after the initial stroke) [2]. However, about 80% of recurrent cardiovascular events could be prevented if major risk factors are eliminated [3]. Close monitoring of the modifiable risk factors for CVD is crucial for the prevention of recurrent CVD events in the primary health care and ambulatory specialist care settings [4]. The widely recognized modifiable risk factors in secondary prevention of CVD include comorbid conditions (arterial hypertension, atrial fibrillation/flutter, carotid artery disease, depression, insomnia, diabetes mellitus, dyslipidemia, abnormal value of BMI (Body Mass Index)), renal and liver impairment, medication use and certain lifestyle characteristics (smoking, alcohol abuse) [5,6,7,8,9,10,11,12,13,14,15,16,17,18,19,20]. It is worthy of note that coincidence of these risk factors is associated with increased rates of recurrent cardiovascular events [21]. Therefore, it is advisable to monitor all of these factors at the same time. The International Classification of Functioning, Disability, and Health (ICF) helps to systematize various clinical data from medical records and present them in a clear graphical form that can be used for comparison and differentiation [22]. The ICF classification makes it possible to combine neurological deficits with activity disturbance in certain activities of everyday life. Moreover, it facilitates the verification and evaluation of the functional state evaluation. Knowledge of the functional abilities of patients, documented in commonly used clinical tests after recoding on the ICF scale, increases the reliability of a comprehensive patient assessment [23]. In practical terms, the ICF classification has so far been used in poststroke patients at the stage of early diagnosis and in the acute stage of stroke treatment [24]. It is of note that, according to the available literature, ICF classification has never been used in secondary prevention to assess CVD risk factors in patients with prior stroke.

### Objective

1. To identify differences in the incidence of risk factors for recurrent CVD events based on clinical type of prior stroke.
2. To use the ICF category charts as a tool for determining differences in the incidence of risk factors for recurrent CVD events in patients with a history of prior ischemic stroke (IS) and intracerebral hemorrhage (ICH).

## 2. Materials and Methods

### 2.1. Study Population/Inclusion and Exclusion Criteria

The study had retrospective design and involved the analysis of risk factors for recurrent CVD events based on data from medical records of patients who were admitted to the Department of Neurological Rehabilitation in the Wiktor Dega Memorial Orthopedic & Rehabilitation Teaching Hospital in Poznań within 14 days of stroke between December 2017 and September 2020. The patients were divided into 2 groups based on prior history of ischemic or hemorrhagic stroke. The study was approved by the Ethics Committee at the Karol Marcinkowski Memorial Medical University in Poznań (Approval No. 810/2017 of 22 June 2017). The study was conducted in accordance with ethical principles for biomedical research as stated in the Declaration of Helsinki. The study was registered in the Clinical Trial Registry: NCT04590287 https://clinicaltrials.gov/ct2/show/NCT04590287 (accessed on 19 October 2020).

Inclusion criteria: (1) history of a new-onset ischemic or hemorrhagic stroke; (2) stroke documented by imaging studies; (3) early hospitalization for stroke (≤14 days after diagnosis) at the department of neurological rehabilitation; (4) complete medical records in respect to the risk factors of interest.

Exclusion criteria: (1) history of multiple strokes; (2) no imaging studies available to confirm the history of stroke; (3) late hospitalization for stroke (>14 days after diagnosis) at the department of neurological rehabilitation; (4) the lack of complete medical records in respect to the risk factors of interest.

### 2.2. Statistical Analysis

The data were analyzed using Statistica version 13.1 software. Descriptive statistics were reported as means, standard deviation (SD), median, minimum, and maximum. Categorical variables were expressed as counts and frequencies. The Shapiro–Wilk test was used to assess the normality of distributions in the test score. Due to non-normally distributed data, the nonparametric U Mann–Whitney test was used to compare outcomes between the IS and HS groups. The chi-squared test was employed to compare differences between groups for categorical variables. *p*-values of less than 0.05 were considered statistically significant. A post hoc evaluation of the power of the study showed following values: hypertension a power of 0.57, coronary heart disease a power of 0.87, chronic heart failure a power of 0.73, type 2 diabetes mellitus (DM) a power of 0.87, carotid artery disease a power of 0.99, epilepsy a power of 0.73.

### 2.3. ICF Profile

The analysis involved the prevalence of risk factors for recurrent CVD events among patients with a prior history of ischemic stroke or hemorrhagic stroke as described in the literature. Specific codes and their respective qualifiers were assigned to each ICF category according to the ICF classification:

The effect of depressive disorders on the risk of a recurrent CVD event was assessed using ICF category b152: emotional functions. The following Beck Depression Inventory (BDI) scores were used to measure the severity of depression [25]: qualifier 0: BDI total score 0 to 11—no depression; qualifier 2: BDI total score 12 to 19—mild depression; qualifier 3: BDI total score 20 to 25—moderate depression; qualifier 4: BDI total score 26 to 63—severe depression.

The effect of sleep disturbance on the risk of a recurrent CVD event was assessed using ICF category b134: sleep functions. The following criteria were used to measure the severity of insomnia [6]: qualifier 0—no sleep disturbance (sleep time 6–9 h); qualifier 4—sleep disturbance (sleep time <6 or >9 h).

The increased risk of CVD related to heart rate (HR) was estimated using ICF category b4100: heart rate. The following criteria were used to quantify heart rate disorders [7]: qualifier 0—HR < 80/min; qualifier 4—HR > 80/min. Heart rhythm disorders were encoded as ICF category b4101: heart rhythm. The following criteria were used [8]: qualifier 0—normal sinus rhythm; qualifier 4—atrial fibrillation.

The effect of carotid artery stenosis on the risk of a recurrent CVD event was assessed using ICF category b4150: functions of arteries. The following criteria were used [9]: qualifier 0—<50% carotid stenosis; qualifier 3—50% to 69% carotid stenosis; qualifier 4—>70% carotid stenosis.

The effect of increased blood pressure (BP) on the risk of a recurrent CVD event was assessed using ICF category b4200: increased blood pressure. The following BP values were used [26]: qualifier 0—BP < 130/80 mm/Hg; qualifier 1—BP > 130/80 mm/Hg; qualifier 2—BP > 140/90 mm/Hg; qualifier 3—BP > 160/90 mm/Hg; qualifier 4—BP > 180/110 mm/Hg.

The effect of liver and renal impairment on the risk of a recurrent CVD event was assessed using ICF category b4301: metabolite-carrying functions of the blood. The following criteria were used to classify renal impairment [27]: qualifier 0—estimated glomerular filtration (eGFR) >90 mL/min/1.73 m^2^; qualifier 1—eGFR 60–89 mL/min/1.73 m^2^; qualifier 2—eGFR 30–59 mL/min/1.73 m^2^; qualifier 3—eGFR 15–29 mL/min/1.73 m^2^; qualifier 4—eGFR < 15 mL/min/1.73 m^2^, and liver impairment [12]: qualifier 0—bilirubin level <2× the upper limit of normal (ULN) and ALT(alanine transaminase)/AST(aspartate transaminaze)/ALP(alkaline phosphatase) <3× ULN; qualifier 4—bilirubin level >2× ULN and ALT/AST/ALP >3× ULN.

Patients receiving anticoagulants have an increased risk of bleeding [13]. This parameter was encoded as ICF category b4302: functions related to the coagulation of blood. If taking VKA (vitamin K antagonist) following values were used: qualifier 0—NO; qualifier 4—YES. If taking NOAC (non-vitamin K antagonist) following values were used: qualifier 0—NO; qualifier 4—YES.

The effect of impaired glycemic control on the risk of recurrent CVD event was assessed using ICF category b5401, carbohydrate metabolism. The following HbA1c (glycated hemoglobin 1c) values were used [28]: qualifier 0—HbA1c < 7%; qualifier 4—HbA1c > 7%.

The effect of LDL-C (low-density lipoprotein cholesterol) levels on the risk of a recurrent CVD event was assessed using ICF category b7302, lipid metabolism. The following LDL-C values were used [29]: qualifier 0—LDL-C <55 mg/dL; qualifier 2—LDL-C 55–70 mg/dL, qualifier 3—LDL-C 71 mg/dL-115 mg/dL, qualifier 4—LDL-C > 116 mg/dL.

Alcohol consumption is an additional risk factor associated with increased risk of a recurrent CVD event. This risk factor was assessed using ICF category e1100, food: alcohol consumption. The following criteria were used [30]: qualifier 0—alcohol intake per day <10 g; qualifier 4—alcohol intake per day >10 g.

The increased risk of CVD related to NSAIDs (nonsteroidal anti-inflammatory drugs) [17] and to smoking [18] was estimated using ICF categories e1101, drugs and e1109, products or substances for personal consumption, respectively. The following criteria were used: qualifier 0—NO; qualifier 4—YES.

Then, a percentage distribution of ICF category qualifiers was graphically represented using color coding in order to highlight differences in the type and incidence of individual risk factors for recurrent CVD events between clinical types of stroke: qualifier 0—no risk factor is present if the percentage distribution ranges from 0% to 4%, color code: dark green; qualifier 1—low prevalence of the risk factor if the percentage distribution ranges from 5% to 24%, color code: light green; qualifier 2—moderate prevalence of the risk factor if the percentage distribution ranges from 25% to 49%, color code: yellow; qualifier 3—high prevalence of the risk factor if the percentage distribution ranges from 50% to 95%, color code: orange; qualifier 4—extremely high prevalence of the risk factor if the percentage distribution ranges from 96% to 100%, color code: red.

## 3. Results

### 3.1. Study Population Characteristics

The study groups comprised 109 patients with a history of prior ischemic stroke (IS) and 80 patients with a history of prior intracerebral hemorrhage (ICH). There was a significant difference between study groups with respect to patients’ age (*p* < 0.001). The median age was 69.3 years in IS group (SD ± 14.8), and 59.4 years in ICH group (SD ± 14.0). There were more women (50.50%) among IS patients, and more men (57.50%) among ICH patients. Furthermore, the incidence of comorbid conditions such as hypertension (*p* = 0.025), coronary heart disease (*p* = 0.001), chronic heart failure (*p* = 0.013), type 2 diabetes mellitus (*p* = 0.002) and carotid artery disease (*p* = 0.013) was significantly higher in the IS group. However, the incidence of comorbid epilepsy was significantly higher in the ICH group (*p* = 0.009). The detailed characteristics of the study population are provided in Table 1.

### 3.2. The Incidence of Risk Factors for Recurrent CVD Events

The incidence of risk factors for recurrent CVD events according to the clinical type of prior stroke is provided in Table 2. The incidence of atrial fibrillation/flutter (*p* = 0.031), >70% carotid artery stenosis (*p* = 0.004), increased blood pressure (*p* = 0.025), increased blood HbA1c (*p* = 0.002), smoking (*p* = 0.026) and NSAID use (*p* < 0.001) was significantly higher in IS patients compared to the ICH group. The incidence of liver function test abnormalities was significantly higher in ICH patients compared to the IS group (*p* = 0.025). There were no significant differences between IS and ICH groups in the incidence of depression, insomnia, heart rate, 50% to 69% carotid stenosis, renal impairment (eGFR < 15 mL/min/1.73 m^2^), VKA/NOAC use, lipid metabolism and alcohol consumption.

### 3.3. CVD Risk Factor Profile According to the ICF Classification

The ICF classification categories presented in Table 3 indicate risk factors which need to be monitored in secondary prevention of CVD. The chart represents percentage distribution of these risk factors according to the clinical type of prior stroke.

For the category “extremely high prevalence”, risk factors such as heart rate and heart rhythm disorders, >70% carotid stenosis, abnormal blood levels of HbA1c > 7%, and NOAC/NSAID use were more frequently observed in patients with a history of IS. The percentage of patients with insomnia and liver impairment was significantly higher in the ICH group. The incidence of depression, increased blood pressure >180/110 mmHg and alcohol consumption was comparable between both groups.

For the category “high prevalence”, risk factor of glomerular filtration rate (eGFR) 15–29 mL/min/1.73 m^2^ was more frequently observed in patients with a history of IS. However, blood pressure >160/90 mmHg and LDL-C levels between 71 and 115 mg% were more common in patients with a history of ICH. The incidence of 50–69% carotid stenosis was comparable between IS and ICH groups.

For the category “moderate prevalence”, risk factors such as blood pressure >140/90 mmHg and LDL-C levels between 70 and 55 mg% were more commonly observed in the IS group compared to ICH group.

## 4. Discussion

Stroke is the second most common cause of death globally and the leading cause of disability among adults in most countries in the world [31]. The increase in the incidence of stroke is strongly correlated with increased lifespan and increased proportion of the population aged 65+ [32]. The results of the research presented in this paper suggest that, for the study population, intracerebral hemorrhage was more commonly observed than ischemic stroke in younger individuals (Table 1). These findings are consistent with the results provided by Yamada et al. [33]. Like Zhang et al. [34], in this paper we demonstrated that the incidence of hypertension, coronary heart disease, chronic heart failure, carotid artery disease and type 2 diabetes mellitus was higher in patients with a history of ischemic stroke (Table 1). However, epilepsy was more commonly observed in patients with a history of intracerebral hemorrhage compared to those with a history of ischemic stroke (Table 1). A study by Feyiss et al. [35] has yielded similar results. They demonstrated that the reported incidence of seizure episodes related to previous stroke varied significantly from 2% for IS patients to 20% for ICH patients.

The presence of modifiable risk factors is associated both with the risk of recurrent CVD events and increased mortality and disability rates [36]. The risk factors and the stratification of CVD risk factors may change over time and should be kept up to date [37]. The incidence of risk factors for recurrent CVD events according to the clinical type of prior stroke in the study population is provided in Table 2 and Table 3. The results showing differences in risk factors between patients with IS and ICH are related to the pathogenetic differences between these groups. These data suggest that atrial fibrillation/flutter and abnormal heart rate were more common in patients with a history of ischemic stroke. Lip et al. in their study [8] have shown that receiving a new-onset AF (atrial fibrillation) diagnosis after ischemic stroke was highly associated with recurrent stroke. Blood pressure >140/90 mmHg is another risk factor more commonly observed in patients with ischemic stroke. Our results are in line with findings by Liu et al. [10] who provided the evidence that blood pressure lowering in patients with a history of stroke reduces the incidence of recurrent CVD events. As we demonstrated in our research, carotid artery stenosis >70% is also more common in patients with a prior history of ischemic stroke. Orrapin et al. [9] found that this risk factor is associated with increased risk of recurrent CVD events. Diabetic patients are more susceptible to atherosclerosis. Wu et al. [14] demonstrated that abnormal levels of glycated hemoglobin are independent predictors for the risk of recurrent ischemic stroke. These findings are consistent with data provided in Table 2 and Table 3. Our results also suggest that the prevalence of smoking and NSAID/NOAC use is higher among patients with a history of ischemic stroke. Active smoking may lead to recurrent CVD events. Cessation of cigarette smoking after an ischemic stroke is associated with decreased 5-year risk of stroke, myocardial infarction or death [18]. According to Gerner et al. [13] and Narum et al. [17], the use of NOAC and NSAID is associated with increased risk of intracerebral hemorrhage.

In our study we demonstrated that sleeping disorders were more common in patients with a prior history of intracerebral hemorrhage than in those with a history of ischemic stroke, which is in line with findings by Cappuccio et al. [6] Abnormal results of certain liver function tests were also more commonly observed in this patient population. A study by Pisters et al. [12] revealed that bilirubin levels >2× upper limit of normal combined with aspartate aminotransferase/alanine aminotransferase/alkaline phosphatase levels >3× upper limit of normal are associated with an increased risk of intracerebral hemorrhage.

The vast majority of studies investigated the impact of a single risk factor [38] or several risk factors [39] on primary prevention of CVD. Risk factors related to the occurrence of qualifier level 4, which are marked in red, require absolute monitoring in the secondary prevention of stroke (Table 3). In patients with a history of ischemic stroke, particular attention should be paid to the occurrence of heart rate and rhythm disturbances, carotid artery stenosis >70%, abnormal HbA1c levels >7% and the use of NOAC/NSAIDs as factors determining the recurrence of a CVD events. On the other hand, after patients have suffered an intracerebral hemorrhage, particular attention should be paid to the occurrence of liver dysfunction and insomnia. The percentage distribution of risk factors for recurrent CVD events according to the ICF classification categories, presented in Table 3, provides information about all recognized risk factors and their prevalence based on the type of prior stroke, gathered in one place and at the same time aims to help healthcare professionals make the best decisions regarding the secondary prevention of cardiovascular disease. Moreover, it enables the users to readily assess individual risk factor categories and create “dynamic charts” to guide clinical decision making.

## 5. Conclusions

(1) The incidence and type of risk factors for recurrent CVD events vary according to the clinical type of prior stroke.
(2) The ICF classification system can be used to evaluate risk factors for recurrent CVD events based on the clinical type of prior stroke.
(3) The use of a single tool that describes multiple risk factors for recurrent CVD events, such as ICF assessment sheet, may increase the effectiveness of secondary prevention interventions, and thus decrease the risk of subsequent cardiovascular events.

## 6. Limitations

The limitations of our study were the lack of analysis of risk factors in relation to personal factors such as age and gender, as these factors are not organized into categories and qualifiers in the current version of the ICF classification. Another issue is the failure to consider BMI as a well-known risk factor for a recurrent CVD events due to the lack of data enabling its calculation based on the available medical documentation. Furthermore, some categories of ICF classification could only be represented as extreme qualifiers (4 or 0) without the possibility of using intermediate values.

## Figures and Tables

**Table 1:** Study population characteristics.

		Ischemic Stroke	Intracerebral Hemorrhage	*p*-Value
Sex	F *n* (%)	55 (50.50%)	34 (42.50%)	0.279
	M *n* (%)	54 (49.50%)	46 (57.50%)	
Age	Mean ± SD (years)	69.3 ± 14.8	59.4 ± 14.0	<0.001 *
	Median (years)	71	61	
	Minmax (years)	51–93	49–84	
Treatment of stroke	Medical (pharmacological) *n* (%)	87 (79.80%)	60 (75.00%)	0.431
	Interventional *n* (%)	22 (20.20%)	20 (25.00%)	
Comorbidities				
Hypertension	*n* (%)	104 (95.4%)	69 (86.30%)	0.025
Coronary heart disease	*n* (%)	26 (23.90%)	6 (7.50%)	0.001
Chronic heart failure	*n* (%)	8 (7.30%)	0 (0.00%)	0.013
Type 2 DM	*n* (%)	36 (33.00%)	11 (13.80%)	0.002
Carotid artery disease	*n* (%)	32 (29.63%)	4 (5.00%)	0.013
Epilepsy	*n* (%)	8 (7.30%)	16 (20.00%)	0.009

Chi-squared test, * Mann–Whitney test, Type 2 DM—type 2 diabetes mellitus, F—females, M—males, *n*—size of the sample, SD—standard deviation, ICH—intracerebral hemorrhage, IS—ischemic stroke.

**Table 2:** The incidence of cardiovascular disease (CVD) risk factors in secondary prevention according to the clinical type of prior stroke.

		Ischemic Stroke	Intracerebral Hemorrhage	*p*-Value
b 152 Emotional functions—Depression [5]	*n* (%)	26 (23.90%)	20 (25%)	0.886
b 134 Sleep functions—Insomnia [6]	*n* (%)	28 (25.70%)	30 (37.50%)	0.082
b 4100 Heart rate [7]	HR > 80/min, *n* (%)	49 (47.10%)	32 (40.00%)	0.355
b 4101 Heart rhythm [8]	Atrial fibrillation/flutter, *n* (%)	32 (29.62%)	13 (16.25)	0.031
b 4150 Functions of arteries [9]	Stenosis 50–69%, *n* (%)	8 (8.00%)	3 (7.90%)	0.984
	Stenosis > 70%, *n* (%)	24 (24.00%)	1 (2.60%)	0.004
b 4200 Increased blood pressure [10]	>140/90 mmHg, *n* (%)	104 (95.40%)	69 (86.30%)	0.025
b 4302 Metabolite-carrying functions of the blood [11,12]	eGFR < 15 mL/min/1.73 m^2^, *n* (%)	30 (27.52%)	10 (12.5%)	0.079
	Bilirubin >2× ULN, ALT/AST/ALP > 3× ULN, *n* (%)	5 (4.60%)	11 (13.80%)	0.025
b 4303 Clotting functions. Functions related to the coagulation of blood [13]	VKA, *n* (%)	22 (20.18%)	11 (13.75%)	0.250
	NOAC, *n* (%)	12 (11.01%)	3 (3.75%)	0.068
b 5401 Carbohydrate metabolism [14]	HbA1c > 7%, *n* (%)	36 (33.00%)	11 (13.80%)	0.002
b 7302 Lipid metabolism [15]	LDL 70–55 mg/dL, *n* (%)	5 (6.25%)	1 (3.03%)	0.488
	LDL 115–71 mg/dL, *n* (%)	31 (38.75%)	16 (48.48%)	0.34
	LDL > 116 mg/dL, *n* (%)	41 (51.25%)	16 (48.48%)	0.789
e1100 Food [16]	Alcohol consumption > 10 g (>1 unit), *n* (%)	5 (4.60%)	5 (6.30%)	0.614
e 1101 Drugs [17]	NSAIDs, *n* (%)	88 (80.70%)	11 (13.80%)	0.001
e1109 Products or substances for personal consumption, other specified [18]	Smoking, *n* (%)	33 (30.30%)	13 (16.30%)	0.026

Chi-squared test ALT—Alanine aminotransferase; AST—Aspartate aminotransferase; ALP—Alkaline phosphatase; CVD—Cardiovascular disease; eGFR—Estimated glomerular filtration rate; HbA1c—Glycated hemoglobin 1c; HR—Heart rate; LDL-C—Low-density lipoprotein cholesterol; NOAC—Non-vitamin K antagonist oral anticoagulants; NSAIDs—Nonsteroidal anti-inflammatory drugs; ULN—Upper limit of normal, VKA—Vitamin K antagonist.

**Table 3:** Percentage distribution of CVD risk factors in secondary prevention according to the clinical type of prior stroke and International Classification of Functioning, Disability and Health (ICF) classification categories.

ICF Category		Impairment/Disability																				
		Complete								Severe		Moderate					Mild				No	
Body Functions	Scoring	PERCENTAGE DISTRIBUTION OF QUALIFIERS																				
b152 Emotional functions [25]	Depression (BDI)	26–63								20–25							10–12			0–9		
	ISCHEMIC STROKE	23.90%				76.10%																
	INTRACEREBRAL HEMORRHAGE	25%				75%																
b134 Sleep functions [6]	Sleep time (h)	<6 and >9																	6 to 9			
	ISCHEMIC STROKE	25.70%				74.30%																
	INTRACEREBRAL HEMORRHAGE	37.50%						62.50%														
b4100 Heart rate [7]	HR	>80/min																	<80/min			
	ISCHEMIC STROKE	47.10%												52.90%								
	INTRACEREBRAL HEMORRHAGE	40%							60%													
b4101 Heart rhythm [8]	Heart Rhythm	Atrial Fibrillation																	Normal sinus rhythm			
	ISCHEMIC STROKE	29.62%					70.38%															
	INTRACEREBRAL HEMORRHAGE	16.25%								83.75%												
b4150 Functions of arteries [9]	Stenosis (%)	>70								50–69										<50		
	ISCHEMIC STROKE	24%				8%		68%														
	INTRACEREBRAL HEMORRHAGE	3%	8%								89%											
b4200 Increased blood pressure [26]	BP (mmHg)	>180/110								>160/90		>140/90					>130/80			<130/80		
	ISCHEMIC STROKE	1%	8.80%								30%		24.50%					35.30%				
	INTRACEREBRAL HEMORRHAGE	1%	15%								25%		25%					32.90%				
b4302 Metabolite-carrying functions of the blood [12,27]	eGFR (mL/min/1.73 m^2^)	<15								15–29		30–59					60–89			≥90		
	ISCHEMIC STROKE	5%	8%								14%		73%									
	INTRACEREBRAL HEMORRHAGE	5%	1%		6%						87.50%											
	Bilirubin (ULN)	>2×																	<2×			
	ALT/AST/Alkaline phosphatase (ULN)	>3×																	<3×			
	ISCHEMIC STROKE	4%	96.00%																			
	INTRACEREBRAL HEMORRHAGE	13.80%								86.20%												
b4303 Clotting functions, Functions related to the coagulation of blood [13]	VKA	YES																	NO			
	ISCHEMIC STROKE	20.18%								79.72%												
	INTRACEREBRAL HEMORRHAGE	13.75%								86.50%												
	NOAC	YES																	NO			
	ISCHEMIC STROKE	11.09%		88.81%																		
	INTRACEREBRAL HEMORRHAGE	4%	96.00%																			
b5401 Carbohydrate metabolism [28]	HbA1 (%)	>7																	<7			
	ISCHEMIC STROKE	33.00%					67.00%															
	INTRACEREBRAL HEMORRHAGE	13.80%								86.20%												
b7302 Lipid metabolism [29]	LDL-C (mg/dL)	>116								115–71		70–55								<55		
	ISCHEMIC STROKE	51.25%													38.75%				6.25%			4%
	INTRACEREBRAL HEMORRHAGE	50%													47.00%							3%
Environmental factors																						
e1100 Food [30]	Alcohol consumption [g]	>10																	<10			
	ISCHEMIC STROKE	4%	96.00%																			
	INTRACEREBRAL HEMORRHAGE	6%	94.00%																			
e1101 Drugs [17]	NSAIDs	YES																	NO			
	ISCHEMIC STROKE	80.70%																	19.30%			
	INTRACEREBRAL HEMORRHAGE	11.30%								88.70%												
e1109 Products or substances for personal consumption, other specified [18]	Smoking	YES																	NO			
	ISCHEMIC STROKE	30.30%					69.70%															
	INTRACEREBRAL HEMORRHAGE	16.30%								83.70%												

ALT—Alanine aminotransferase; AST—Aspartate aminotransferase; ALP—Alkaline phosphatase; BP—Blood pressure; CVD—Cardiovascular disease; eGFR—Estimated glomerular filtration rate; ICF—International Classification of Functioning, Disability and Health; HbA1c—Glycated hemoglobin 1c; HR—Heart rate; LDL-C—Low-density lipoprotein cholesterol; NOAC—Nonvitamin K antagonist oral anticoagulants; NSAIDs—Nonsteroidal anti-inflammatory drugs; ULN—Upper limit of normal; VKA—Vitamin K antagonist.

## Data Availability

The data presented in this study are available on request from the corresponding author. The data are not publicly available due to ethical restrictions.
